# Can Dietary Patterns Impact Fertility Outcomes? A Systematic Review and Meta-Analysis

**DOI:** 10.3390/nu15112589

**Published:** 2023-05-31

**Authors:** Hugo G. Winter, Daniel L. Rolnik, Ben W. J. Mol, Sophia Torkel, Simon Alesi, Aya Mousa, Nahal Habibi, Thais R. Silva, Tin Oi Cheung, Chau Thien Tay, Alejandra Quinteros, Jessica A. Grieger, Lisa J. Moran

**Affiliations:** 1 Department of Obstetrics and Gynaecology, Monash University, Melbourne, VIC 3800, Australia; hugowinter97@gmail.com (H.G.W.); daniel.rolnik@monash.edu (D.L.R.); ben.mol@monash.edu (B.W.J.M.); 2 Aberdeen Centre for Women’s Health Research, Institute of Applied Health Sciences, School of Medicine, Medical Sciences and Nutrition, University of Aberdeen, Aberdeen AB24 3FX, UK; 3 Monash Centre for Health Research and Implementation (MCHRI), Monash University, Melbourne, VIC 3800, Australia; sophia.torkel@monash.edu (S.T.); simon.alesi1@monash.edu (S.A.); aya.mousa@monash.edu (A.M.); jillian.tay@monash.edu (C.T.T.); 4 Adelaide Medical School, The University of Adelaide, Adelaide, SA 5005, Australia; nahal.habibi@adelaide.edu.au (N.H.); tinoi.cheung@adelaide.edu.au (T.O.C.); quinteros.thais1@gmail.com (A.Q.); jessica.grieger@adelaide.edu.au (J.A.G.); 5 Robinson Research Institute, The University of Adelaide, Adelaide, SA 5005, Australia; 6 Postgraduate Program in Endocrinology and Metabolism, Universidade Federal do Rio Grande do Sul, Porto Alegre 90010-150, Brazil; thaisrasia@gmail.com

**Keywords:** diet, fertility, pregnancy, live birth, infertility, Mediterranean diet, diet pattern, whole diet, systematic review

## Abstract

There are conflicting results on the effect of diet on fertility. This study aimed to assess the effect of different dietary patterns on fertility outcomes in populations who conceive spontaneously and those requiring assisted reproductive technology (ART). A systematic search and meta-analysis were performed for studies investigating dietary patterns or whole diets in reproductive aged women requiring ART or conceived naturally. Outcomes were live births, pregnancy rates and infertility rates. In amount of 15,396 studies were screened with 11 eligible studies. Ten different diet patterns were grouped broadly into categories: Mediterranean, Healthy or Unhealthy. For the Mediterranean diet, on excluding high risk-of-bias studies (*n* = 3), higher adherence was associated with improved live birth/pregnancy rates in ART [OR 1.91 (95% CI 1.14–3.19, *I*^2^ 43%)] (*n* = 2). Adherence to various Healthy diets was associated with improved ART outcomes (ProFertility diet and Dutch Dietary Guidelines) and natural conception outcomes (Fertility diet). However, due to the variability in Healthy diets’ components, results were not pooled. Studies demonstrated preliminary evidence for the role of dietary patterns or whole diets in improving pregnancy and live birth rates. However, due to heterogeneity across the literature it is currently unclear which diet patterns are associated with improvements in fertility and ART outcomes.

## 1. Introduction

Infertility, defined as the inability to conceive after 12 months of regular unprotected sexual intercourse, is a growing health concern [1]. According to the World Health Organization (WHO), infertility is estimated to affect 48 million couples globally; this equates to 15% of couples trying to conceive [2]. The rise in use of assisted reproductive technology (ART) including in vitro fertilization (IVF) and intracytoplasmic sperm injection (ICSI), now accounts for up to 1 in 20 births in developed nations [3,4,5]. In developing nations, many with higher infertility rates, equitable access to ART is a growing area of concern [6]. Despite the prevalent use of ART and advances in this area, success rates remain low, with an average of one in five cycles ending in a live birth [7]. ART carries significant financial and emotional strain, and research into potential risk factors for infertility and associated management strategies is of compelling importance.

Over the years, much research has explored risk factors for infertility, both non-modifiable, such as age, congenital abnormalities or tubal damage, and modifiable such as smoking, excessive weight, alcohol consumption and physical activity [8,9]. The effect of dietary intake, despite being well studied in many other non-communicable diseases, has only recently been explored with regards to infertility and ART, and reviews or guidelines are mostly limited to polycystic ovary syndrome (PCOS) [10]. Beyond conception, healthy lifestyle changes for these patients may also carry lifelong benefits in many other health domains such as cardiovascular health or cancer [11,12].

Prior research in this area has predominantly focused on single nutrients or food groups across a variety of reproductive health outcomes. However, foods are not consumed in isolation and are consumed habitually in combination with others as whole diets or dietary patterns. Dietary patterns or whole diet approaches may produce additive or synergistic benefits which may have larger impacts than single nutrients alone [13]. Dietary pattern or a holistic whole diet analysis has become increasingly commonplace in nutritional epidemiology and aims to quantify holistic, habitual dietary intake as a single exposure [13].

Despite multiple studies conducted in the last decade on whole diet and fertility outcomes, such as pregnancy rates, live births, infertility rates and an array of surrogate markers including, embryo quality, endometrial thickness, hormone levels and embryo yield, results have been inconsistent across a variety of populations [14,15]. Previous systematic reviews have explored results in narratively synthesised analysis, or among populations requiring ART only [16,17,18]. The primary aim of this review is to systematically review whole diet/dietary patterns and the relationship to pregnancy rates, live birth rates and infertility rates across both natural conception and ART cohorts. This review also aims to evaluate surrogate markers of fertility; for example, embryo quality or endometrial thickness, commonly used in research as primary endpoints, to explore the potential mechanism of impact.

## 2. Materials and Methods

### 2.1. Registration

The review protocol was developed in line with the Preferred Reporting Items for Systematic reviews and Meta-Analysis Protocols (PRISMA-P) statement [19] and registered with PROSPERO (PROSPERO Registration CRD42021238676). This review was performed as a follow-on review from a scoping review of diet and female fertility prospectively registered with Open Science Framework (OSF) (10.17605/OSF.IO/FBV6W) [20].

### 2.2. Selection Criteria

The Participants, Intervention, Comparison, and Outcome (PICO) framework was used to prospectively design the review protocol. Studies were included if participants were women of childbearing age intending to become pregnant through natural conception or ART. Exclusion criteria were ART studies not of IVF or ICSI (e.g., surrogacy), women outside reproductive age (<18 or >49 years), animal studies, studies of underweight patients or of hereditary disorders in one or both partners (e.g., Fragile X, Turner syndrome, thalassemia). The exposure assessed was whole diets, whereas studies of dietary weight loss or individual micro or macronutrients were excluded.

Whole diet was determined through a priori or posteriori dietary pattern analysis. A priori analysis is based on predetermined diet patterns, and posteriori is based on dietary patterns evident from assessment of the data [13]. Exclusion criteria were randomised controlled trials, narrative reviews or case reports. No language restrictions were applied.

### 2.3. Outcomes

Primary outcomes were live birth (birth of a neonate at or after 24-weeks’ gestation), clinical pregnancy (presence of an intra-uterine pregnancy confirmed by ultrasound at 6 or more weeks of gestation) and biochemical pregnancy (positive urine ß-hCG) [21]. Live births were the outcome of choice for the meta-analysis, and where not published, clinical pregnancies and then biochemical pregnancies were chosen for pooled analysis. Secondary outcomes of interest included surrogate markers of fertility (i.e., oocyte yield, embryo quality, fertilization rate or biochemical markers). Markers were compared with primary outcomes in an attempt to explore the mechanism through which diet may influence fertility.

### 2.4. Search Strategy

**Figure 1:**
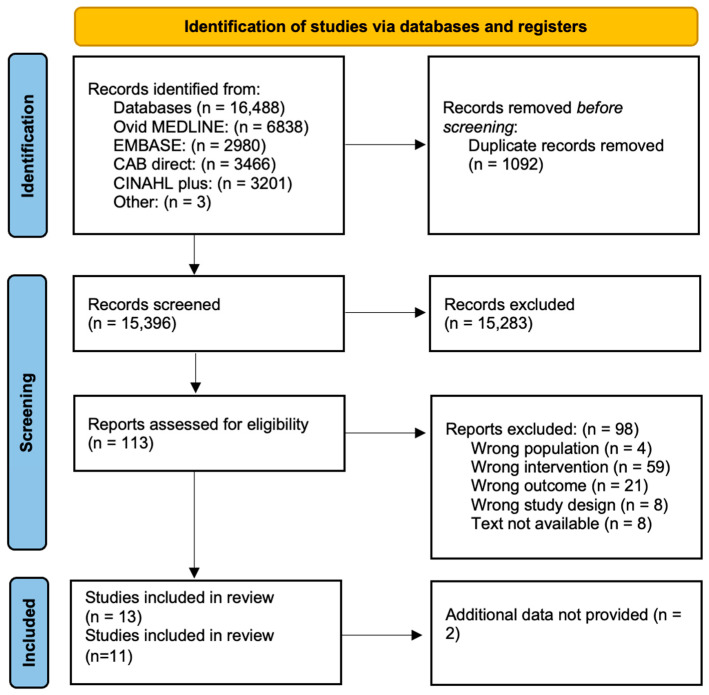
PRISMA diagram for the literature search process.

The search was performed on the 21 September 2021 on the following electronic databases, Ovid MEDLINE, EMBASE, CAB direct and CINAHL Plus (Figure 1). The search strategy is detailed in the Supplementary Material. Title and abstract screening and full text screening were conducted in duplicate using Covidence by authors HW, ST, SA, NH, TS, TC, CT, AQ, JG, and LM independently. Any discrepancies on eligibility were resolved in discussion with a third author until a consensus was reached. References of relevant studies and similar reviews were manually searched for identifying additional relevant articles.

### 2.5. Data Extraction and Quality Assessment

For the studies included, the following data were extracted: author, country, year, study design, study population, participants demographics, dietary assessment method (i.e., Food Frequency Questionnaire (FFQ), 24 h recall or food record); dietary pattern identification (i.e., a priori or posteriori); name and dietary pattern components; data analysis approach; whole diet identified and their main outcomes. If key information was unavailable, the corresponding authors were emailed for clarification twice. Two authors independently assessed methodological quality in duplicate, according to the Risk Of Bias In Non-Randomised Studies of Interventions (ROBINS-I) [22,23].

### 2.6. Classification of Dietary Intake

In each study, authors assigned various foods or food groups, high or low consumption in each of the diets. For studies evaluating whole diet using a priori methods, dietary components specified by each study were used. In studies using posteriori methods, correlation coefficients of <−0.25 or >0.25 were used as cut offs to determine weightings for low and high consumption foods, respectively. Diets were grouped into the common patterns; “Mediterranean” diet, and diets considered by the original authors to be “Healthy” or “Unhealthy” (Table 1).

### 2.7. Statistical Analysis

Random effects model meta-analyses [24] were performed in cases where two or more studies reported data on similar outcomes across a high and low diet adherence group, and were clinically and methodologically similar (as was the case for the various Mediterranean diets). Forrest plots without pooled effect were generated where dietary components were dissimilar (as was the case with Healthy diets). Live births were preferentially used for calculations, in keeping with recommendations from the fertility Core Outcomes Set [25]. However, where studies did not report live births, composites were made by substituting clinical pregnancy (the preference) or biochemical pregnancy, in forest plots and meta-analysis. Similar approaches have been taken in the literature [26].

Pooled effect of crude odds ratio (OR) was conducted using the DerSimonian and Laird methods to estimate between study variance, and the adjusted OR were calculated using generic inverse-variance meta-analysis [27]. Originally reported adjusted risk ratios were converted to odds ratios using the method proposed by Zhang and Yu, to perform the meta-analyses of estimates adjusted for confounding [28]. Heterogeneity was assessed using Cochran’s Q statistic and *I*^2^ with 25%, 50% and 75% defined as low, moderate and substantial heterogeneity [29]. Where pooled effects were produced, heterogeneity was explored through subgroup analyses, according to outcomes, study design and a priori vs. posteriori. Sensitivity analysis was conducted using both leave-one-out analysis and excluding studies at high risk of bias [30]. In addition, due to the tendency for OR to overestimate relative risk (RR), crude pooled RR were calculated on data where pooled OR had also been calculated [28]. Evidence of publication bias was assessed using the Eggers’ regression test of funnel plot asymmetry [31] and in using funnel plots of comparisons that included more than 10 studies [32]. Narrative synthesis was performed where study methods and outcomes were not quantitatively comparable. Statistical analysis was carried out using R (version 4.2.1, 2022, The R Foundation for Statistical Computing, Vienna, Austria), using the package metafor [30,33].

## 3. Results

### 3.1. Description of Studies

The PRISMA flow diagram is provided in Figure 1 [34]. An amount of 16,488 articles were generated by the search, of which 1092 were duplicates. Among 15,396 abstracts and titles screened, 113 proceeded to full text review and 13 studies met the eligibility criteria, with 11 providing sufficient data. Corresponding authors of the study by Twigt et al. and Gaskins et al. provided additional information [21,35,36,37]. Studies by Jahangirifar et al. and Diba-Bagtash et al. were excluded where additional information required to meet the PICO criteria (Population, Intervention, Control and Outcome) was not provided (complete outcome data was not supplied for both studies) [38,39].

### 3.2. Study Characteristics

Of the 11 included studies, there were 10 cohort studies [14,15,35,40,41,42,43,44,45], and 1 nested case-control [46] (Table 2). Studies were conducted in seven countries: four in the United States [15,21,44,45], and two in the Netherlands [14,35], Spain [46], Italy [43], Greece [40], Japan [41] and China [42]. Four studies examined the effect of diet on natural conception [15,44,45,46], and the remaining seven studies focused on couples undergoing ART. No studies included both ART and natural conception. The study sample sizes ranged from 140 to 590 participants in ART studies and between 131 and 17,544 participants in natural conception, totalling 33,055 women included across all studies. Populations showed varied baseline characteristics and confounding variables such as age, smoking, BMI, supplement use or infertility diagnosis (i.e., female factor, male factor, unknown) [14,15,21,35,38,40,41,42,43,44,46]. ART protocols generally were minimally documented [14,15,21,35,38,40,41,42,43,44,46] and some ART studies limited their participants to those undergoing their first cycle of ART [35,40,41], while others did not [14,21,42,43].

In the 11 observational studies, dietary pattern adherence was gauged through a priori method in 8 [15,21,35,40,42,43,44,45] and posteriori in 3 [14,41,46] studies. Food frequency questionnaires was used in 10 studies [14,15,21,35,40,41,42,43,44,46] and serial 24 h food recall phone interviews in 1 study [45]. Three studies examined multiple primary outcomes (i.e., live births and clinical or biochemical pregnancies) [21,40,43]. A range of secondary outcomes (surrogate markers) were included in eight studies [14,21,40,41,42,43].

### 3.3. Quality Assessment

Quality assessment found that eight studies were of moderate risk of bias [14,15,21,35,40,41,44,46] and three were of serious risk of bias [42,43,45]. Across the seven ROBINS I domains, those frequently at moderate or serious risk of bias were for confounding, selection bias and classification of intervention (Table 3).

### 3.4. Primary Outcomes

Three ART studies, which included six individual dietary patterns, reported multiple primary outcomes. For all six dietary patterns, there were consistent association in effects’ direction across primary pregnancy outcomes (Table 4) [21,40,45]. The dietary patterns did, however, trend towards larger effects as pregnancy progressed from biochemical pregnancy to clinical pregnancy through to live birth. For example, the ProFertility diet, was associated with a 48%, 53% and 70% increase in rates of biochemical, clinical pregnancy and live births, respectively. Due to the consistency across outcomes, meta-analysis was conducted using a combination of primary outcomes.

### 3.5. Mediterranean Diets

Five studies examined the association between the Mediterranean diet and ART outcomes in 1342 women [14,21,40,42,43]. Two studies examined the Mediterranean diet and natural conception in 13,226 women [44,46]. Mediterranean diet components were similar across observational studies, with high consumption of fruit, vegetables, fish, nuts, healthy fats and low intake of meats and unhealthy fats (Table 1). Minor variation did exist in intake of grains, alcohol and dairy across studies.

Adherence to the Mediterranean diet was positively associated with improved ART outcomes (biochemical pregnancies and live births) in three of the five studies [14,21,40]. Vujkovic et al. reported an OR of 1.4 (95% CI 1.0–1.9) for biochemical pregnancies among couples with high adherence but not for female diet alone [OR 1.27 (95% CI 0.48–3.38)]. In a post hoc analysis comparing live births in low adherence quartile to pooled second, third and fourth quartiles, Gaskins et al. reported an RR of 1.41 (95% CI 1.10–1.80). The third study, by Karayiannis et al. reported an RR of 3.12 (95% CI 1.39–7.03) across highest to lowest tertile in live births. Two studies examined the Mediterranean diet and natural conception. One study found no association between an “alternative Mediterranean diet” and risk of pregnancy loss (RR 1.02, 95% CI 0.98–1.05) [44]. In contrast, a nested case-control study found Mediterranean diet adherence during university years was associated with lower odds of seeking fertility treatment later in life [OR 0.56 (95% CI 0.35–0.95)] [46].

**Figure 2:**
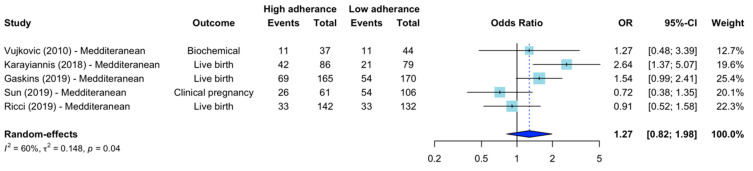
Forest plot of studies comparing groups of high and low adherence to Mediterranean diets and ART outcomes. Random effects model meta-analysis of crude OR, Vujkovic et al. highest to lowest tertile [14], Karayiannis et al. highest to lowest tertile [40], Ricci et al. [43], Gaskins et al. [21], highest to lowest quartile, Sun et al. higher to lower half [42]. OR, odds ratio. CI, confidence interval.

The pooled crude OR for the association of Mediterranean diet with ART outcomes (live birth and clinical and biochemical pregnancy) was 1.27 (95% CI 0.82–1.98, *I*^2^ 60%) (Figure 2). On subgroup analysis by dietary measurement (a priori, posteriori or intervention) or outcomes (live birth or clinical or biochemical pregnancy), there was no differences in the pooled effect, and moderate heterogeneity was maintained (Table 5). On sensitivity analysis excluding studies of serious risk of bias, the Mediterranean dietary pattern was significantly associated with improved ART outcomes [OR 1.91 (95% CI 1.14–3.19, *I*^2^ 43%)]. The leave-one-out analysis did not change the overall effect observed, nor did calculating the pooled RR instead of OR, or the OR adjusted for confounders instead of crude OR (Supplementary Figures S5 and S6).

### 3.6. Healthy Diets

Four studies in ART [14,21,35,41] and three studies in natural conception [15,44,45] populations, identified various dietary patterns considered to be Healthy, i.e., generally high in vegetables and fruit, but with a mix of other food groups included (Table 1). For example, meat was considered healthy in some diets [35,41], but unhealthy in others [14,21]. Similar differences were seen in consumption of grains and oils, but also fruits and vegetables [15,44,45]. Due to this significant variability in dietary components, pooled effects were not calculated.

**Figure 3:**
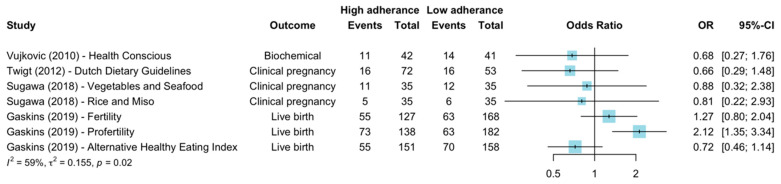
Forest plot of studies comparing groups of high and low adherence to various Healthy diets and ART outcomes. Random effects meta-analysis of crude OR, Vujkovic et al. [14] and Twigt et al. [35] highest to lowest tertile, Sugawa et al. [41] and Gaskins et al. [21] highest to lowest quartile. OR, odds ratio. CI, confidence interval.

Of the four ART studies examining seven different Healthy diets, two reported improvements in clinical pregnancies and live births (Figure 3) [21,35]. The first, the Dutch Dietary Guidelines, high in fruit, vegetables, meat, fish, healthy fats and whole grains, demonstrated an adjusted 65% increase in odds of achieving clinical pregnancy with each incremental increase in adherence (scored across six domains, with a total score of 0–6) [OR 1.65 (95% CI 1.08–2.52)]. Authors were contacted and provided additional information in order to group diet scores of 0, 1 and 2 as low adherence, and scores 4, 5 and 6 as the high adherence group with groups approximately one-third of participants. When effects were recalculated comparing higher and lower tertiles/adherence groups, results were highly variable [crude OR 0.66 (95% CI 0.29–1.48) and adjusted OR 2.94 (95% CI 0.88–9.79)]. The second diet, ProFertility diet, high in low pesticide fruits and vegetables, fish, soy and supplements, reported significant improvements across multiple outcomes, biochemical pregnancy, clinical pregnancy and live births, RR 1.48, RR 1.53, RR 1.70 (*p* < 0.001 for all outcomes), respectively (Table 3) [21]. The other dietary patterns examined by these four studies (Health Conscious-low processed, Vegetables and Seafood, Rice and Miso, Fertility, alternative Healthy Eating Index (aHEI)) were not associated with primary outcomes [14,21,41].

Three studies examined diet and natural conception, one study of pregnancy loss and two of infertility [15,44,45]. One of the two studies reported improvements in infertility rates in natural conception. Chavarro et al. reported those adherent to the Fertility diet—high in unsaturated fats, vegetables, high fat dairy and supplements—had 66% lower odds of ovulatory infertility (RR 0.34, 95% CI 0.23–0.48) and 27% lower risk of infertility due to other causes (RR 0.73, 95% CI 0.57–0.95) [15]. In the study by Gaskins et al., the same diet was then studied alongside the alternative Mediterranean and aHEI diet and found no significant association between any of the three diets and pregnancy loss [44]. This was the same Fertility diet that was later shown to have no association with ART outcomes [21].

### 3.7. Unhealthy Diets

Three studies considered typical Unhealthy diets and fertility outcomes. The study by Sugawa et al. evaluated the effects of a Western Diet on clinical pregnancy rates, finding no significant association (OR 0.84, 95% CI 0.23–3.11) [41,47]. In the study from Toledo et al., no significant association was found between the Western-type diet and odds of seeking help for infertility (OR 0.91, 95% CI 0.66–1.24) [46]. Hartman et al. reported that a high energy-density diet, high in saturated fats, low in fruit vegetables and grains, was associated with a 70% reduction in clinical pregnancy rates (OR 0.30, 95% CI 0.11–0.81) when comparing the highest to the middle adherence tertile [45]. This finding was, however, not statistically significant across highest to lowest tertiles of pregnancy rates (OR 0.69, 95% CI 0.30–1.61), or live births (OR 0.69, 95% CI 0.30–1.61).

### 3.8. Secondary Outcomes

Of the seven studies examining ART, six included analyses of surrogate markers of ART success [14,21,40,41,42,43]. In combination, more than 30 separate markers were examined including biochemical markers, fertilization rates of the ovum and embryo quality (Table 2). Studies included between 2 and 15 surrogate markers. The choice of surrogate markers varied dramatically, with only two markers being included in over two studies (total oocyte yield and number of high-quality embryos).

Of the six studies, only two reported significant association between diet and surrogate markers. These were red blood cell folate and vitamin B6 levels with Mediterranean diet and Health-conscious diet [14], and number of embryos and fertilized oocytes available with adherence to Mediterranean diet [42]. None of these findings were reproduced by other studies. In addition, these markers were not significantly associated with any primary outcomes (i.e., live births), and diets that were associated with increased pregnancy and live birth rates; for example, the study by Karayiannis et al. had no significant association with any of the 10 surrogate markers measured [40].

### 3.9. Publication Bias

**Figure 4:**
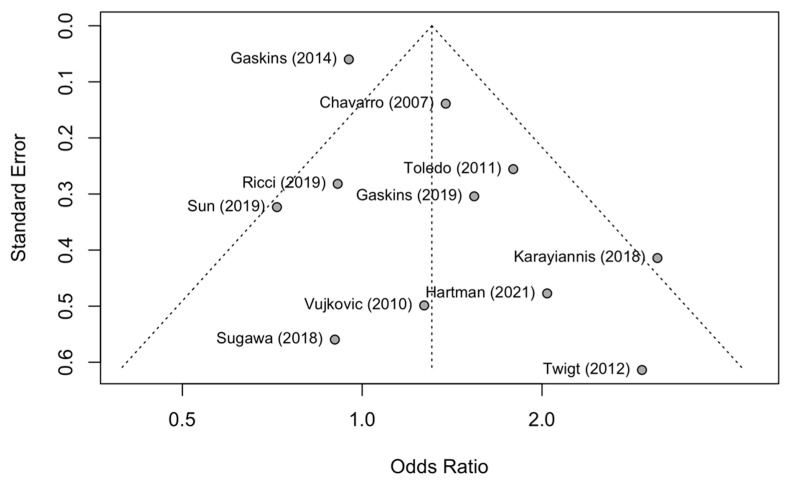
Funnel plot of both natural conception and ART for the dietary comparison on primary outcomes. Studies by Vujkovic et al. (2010) [14], Gaskins et al. (2019) [21] use published results of Mediterranean diet. Studies by Sugawa et al. (2018) [41] uses Vegetables and Seafood diet, Gaskins et al. (2014) [44] and Chavarro et al. (2007) [15] use Fertility diet. All other studies, Twigt et al. (2012) [35], Karayiannis et al. (2018) [40], Sun et al. (2019) [42], Ricci et al. (2019) [43], Toledo et al. (2011) [46] and Hartman et al. (2021) [45] assessed single diet patterns, which are included in the funnel plot. Studies by Gaskins et al. (2014) [44], Toledo et al. (2011) [46], Chavarro et al. (2007) [15], Hartman et al. (2021) [45] published effects of infertility rates/pregnancy loss in natural conception, reciprocals of published results are presented here in funnel plot.

Assessment of publication bias was performed for the comparison on primary outcomes. There was some evidence of publication bias, as shown in the funnel plot (Figure 4), with the Egger’s test suggesting possible asymmetry (*p* = 0.05). We note, however, that this may represent heterogeneity in study designs, rather than publication bias [48].

## 4. Discussion

### 4.1. Principal Findings

We report here the first systematic review assessing dietary patterns and whole diets and the relationship with fertility outcomes in populations conceiving spontaneously and in those requiring ART. We identified four studies of natural conception and seven of ART, broadly covering three dietary patterns, namely Mediterranean, Healthy and Unhealthy. Most studies included were cohort studies with significant risk of (measured or unmeasured) confounding. The literature in this area is heterogenous and difficult to interpret.

High adherence to the Mediterranean diet was found to potentially increase rates of pregnancy and live birth by approximately one-third, which increased to more than two-thirds on exclusion of studies of serious risk of bias. In the limited studies of natural conception (*n* = 2), conflicting results showed Mediterranean diet adherence halved rates of infertility in one study [46], but was unrelated to pregnancy loss in another [44]. Heterogeneity here, may be explained by differences in primary outcome (i.e., infertility rates or pregnancy loss), diet composition (Table 1), study design (i.e., case control or cohort) and populations (Table 2).

A variety of Healthy diets were identified and generally differed in terms of the included food groups, and none were reproduced across the other fertility literature. In ART studies, high adherence to two types of Healthy diets (ProFertility [21] and Dutch Dietary Guidelines [35]) were related to improvements in pregnancy rates and live births rates, by roughly two-thirds each. Studies of natural conception were also conflicting and reported that the Fertility diet reduced infertility risk by between one-third to a half [15]; however, later studies showed no impact on pregnancy loss in natural conception or any ART outcomes [21,44], with similar differences in methodology between studies as seen in natural conception and the Mediterranean diet (Table 2).

Studies of Unhealthy or Western diets failed to show significant association with pregnancy outcomes across highest to lowest adherence groups. Across the variety of surrogate markers reported (e.g., biochemical markers, ovum fertilization or embryo morphology), there was no consistency between which markers were reported across studies, nor were markers correlated with primary outcomes within studies (i.e., pregnancy rates or live births).

### 4.2. Strengths and Limitations

A strength of this study is the focus on whole dietary assessment. Compared to traditional approaches focusing on single nutrients or food groups, whole diet exploration through dietary pattern analysis is able to detect cumulative impacts of several dietary components at once [13]. This is better able to account for the relationship and interaction between different foods [13]; for example, the small improvements seen with each component of the ProFertility diet which are then built up to a larger cumulative impact on primary outcomes [21]. We also report on both clinical primary endpoints alongside surrogate markers to explore possible mechanisms of impact. Limitations to this review include the largely observational nature of the literature in this area and the associated risk of unmeasured confounding. Other limitations, contributing to significant heterogeneity, include variability in clinical populations and diets, both geographically and culturally, as well as in their methodology. These concerns and their implications have already been highlighted in other research in the area [49]. We also note limitations inherent to FFQs such as risk of recall and social approval bias [50]. Lastly, selection bias remains a difficult factor to control in this setting, as due to the evident impact of diet on natural conception, it remains possible that couples with healthier diets undergoing ART may have differing underlying causes of their infertility than those with unhealthy diets.

### 4.3. Clinical and Research Implications

Many studies have published relationships of individual food groups, similar to those constituting the Mediterranean diet or Healthy diets, such as fish, fatty acids, whole grains, soy or antioxidants with fertility outcomes [51,52,53,54,55]. In vitro studies have shown Omega 3 fatty acids, found in fish, to improve embryo morphology [56] and antioxidants to improve embryo development [57]. Studies of soy intake similarly demonstrated improved ART outcomes, possibly through the effects of isoflavones on endometrial thickness [58]. Of interest, there was no association in this study between dietary patterns and well-established surrogate markers, such as total oocyte yield or fertilization rate, despite the significant effects of diet on primary pregnancy endpoints. This highlights the gap in knowledge of the biological mechanisms by which diet influences ART outcomes. While this research question is important, results must be viewed with caution, based on the lack of correlation between these markers and clinically important fertility outcomes (i.e., pregnancy, infertility and live birth rates).

There have been multiple systematic reviews in this area over the last five years [16,17,18,59]. We have extended the literature by including both natural conception and ART populations, quantitatively comparing similarities and differences of individual diets, performing meta-analyses adjusted for important confounders, sensitivity analysis including study quality, and investigating surrogate markers and their relation to clinical outcomes. Three prior systematic reviews reported significant methodological heterogeneity and variable results and concluded that diet did not have an appreciable association with ART outcomes [17,18,59]; two of these based this on qualitative analysis alone [17,59]. The third published a meta-analysis of all Healthy and Mediterranean diets grouped as one exposure [18], which may not be appropriate due to the variable nature of the different diets. The review also combined published and unpublished results from studies that measured over different parameters. Specifically, some studies compared highest and lowest adherence groups [14,21,40,42,43], while the study by Twigt et al. compared incremental increases in diet adherence with data recalculated for comparability with the other studies [35].

There have been two interventional studies worth noting in this area [60,61]. The first, by Kermack et al. was a blinded RCT of 111 women undergoing ART to “Mediterranean diet” supplements and placebo [62]. While the study was well conducted and found multiple improvements in various surrogate markers, it was underpowered for outcomes of pregnancy rates or live births. In addition, the surrogate markers reported in the study have not been reported in any of the observational studies reported in this review, which adds uncertainty to their clinical relevance. The second study, an unblinded RCT by Alibeigi et al., reported improved clinical pregnancy rates with adherence to Iranian Traditional Medicine-Based Diet, [OR 4.29 (95% CI 1.31–14.04)]. The diet, however, was poorly defined with advice to exclude “cold or sour foods” or “foods that produce melancholic sputum” and was additionally at serious risk of bias across multiple areas.

The evidence regarding the effects of diet on fertility remains inconsistent and our review emphasises the need for large well-conducted RCTs to enhance the evidence base for future nutrition recommendations or guidelines for fertility. Despite the inherent difficulties in designing dietary interventions for studies, recent high quality RCTs have succeeded in similar areas. For example, a large RCT showed the Mediterranean diet during pregnancy significantly reduced odds of low birth weight by 42% (OR 0.58, 95% CI, 0.40–0.84) [63]. The potential clinical benefits of this research are far reaching both in fertility outcomes and more broadly. Furthermore, while changes in diet are challenging, they are a low-cost intervention, accessible to all, with well-established, long-lasting health benefits in many domains of human health [64,65].

## 5. Conclusions

Dietary pattern studies are inconsistent for their effect on a range of reproductive outcomes. Most promising appears to be preliminary evidence that the Mediterranean diet improves the probability of achieving reproductive outcomes including pregnancy and live birth as well as decreasing the risk of pregnancy loss. Due to variable results, diets and methodology, it is currently unclear if and which diet patterns are responsible for improvements in fertility and ART outcomes.

## Figures and Tables

**Table 1:**
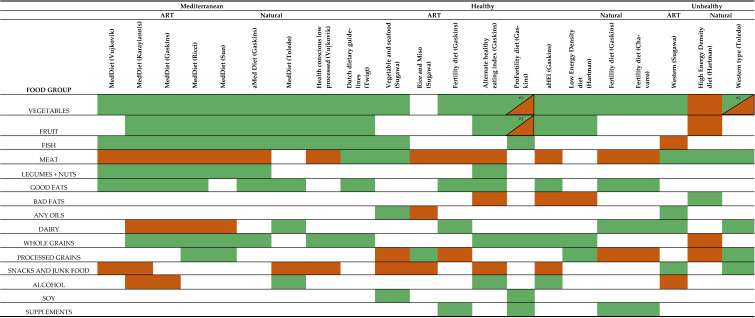
Individual diet components.

Table of individual diet components. High consumption food groups for each diet patterns are highlighted green, low consumption foods are in red. For studies using a priori method of dietary pattern analysis, original food frequency questionnaires (FFQ) were used. Studies utilising posteriori methods correlation coefficients of <0.25 and >0.25 were used as cut off points. MedDiet—Mediterranean diet, aHEI—Alternative Healthy Eating Index 2010. *1 ProFertility diet (Gaskins): high intake of low pesticide fruits and vegetables, low intake of high pesticide fruits and vegetables. *2 Western type diet (Toledo): high intake of potatoes, low intake other vegetables.

**Table 2:** Characteristics of included studies.

Author	Country	Study Design	Population	Diet Assessment Method	Diets Identified	Primary Outcomes	Secondary Outcomes
Vujkovic et al., 2010 [14]	The Netherlands	Cohort study	161 couples ART	Self-reported FFQ + baseline questionnaire Validated Posteriori	Health conscious–low processed Mediterranean	Biochemical pregnancy	RBC folate, folate, vitamin B6, vitamin B12, tHcy
Twigt et al., 2012 [35]	The Netherlands	Cohort study	199 women ART	Self-reported FFQ + baseline questionnaire, professionally checked a priori	Dutch Dietary Guidelines	Clinical pregnancy	-
Karayiannis et al., 2018 [40]	Greece	Cohort study	244 women ART	Self-reported FFQ + baseline questionnaire Validated a priori	Mediterranean	Biochemical pregnancy Clinical pregnancy Live birth	Total oocyte, mature oocyte M2, Fertilized embryo, day 3 FSH, day 3 LH, day 3 oestradiol, baseline AMH, fertilization rate, embryos produced, high quality embryos, number of embryos transferred
Sugawa et al., 2018 [41]	Japan	Cohort study	140 women ART	Self-reported FFQ + baseline questionnaire Validated posteriori	Vegetables and Seafood Western Rice and Miso	Clinical pregnancy	Total oocyte yield, Veeck’s criteria
Gaskins et al., 2019 [21]	USA	Cohort study	357 women ART	Self-reported FFQ + baseline questionnaire Validated a priori	Mediterranean diet Alterative Healthy Eating Index 2010 Fertility diet ProFertility diet	Biochemical pregnancy Clinical pregnancy Live birth	Oestradiol trigger levels, endometrial thickness, total oocyte yield, mature oocytes M2, fertilized embryos
Sun et al., 2019 [42]	China	Cohort study	590 women 166 in final analysis ART	Self-reported FFQ + baseline questionnaire Not validated a priori	Mediterranean diet	Clinical pregnancy	Endometrial thickness, Gn duration, total oocyte yield, mature oocytes M2, fertilization rate, high quality embryos, number of embryos transferred, basal FSH, basal LH/oestradiol/progesterone, HCG, day 3 LH/oestradiol/progesterone, AFC, number embryos available, number of ET cycles.
Ricci et al., 2019 [43]	Italy	Cohort study	414 women ART	Self-reported FFQ + baseline questionnaire Validated a priori	Mediterranean diet	Clinical pregnancy Live birth	FSH level, AMH level, good quality oocytes, good quality embryos, embryo transfer
Toledo et al., 2011 [46]	Spain	Nested case-control study	485 cases 1669 controls Natural Conception	Self-reported FFQ + baseline questionnaire Validated posteriori	Mediterranean diet Western diet	Consulted a doctor for difficulty conceiving	-
Chavarro et al., 2007 [15]	USA	Cohort study	17,544 women Natural Conception	Self-reported FFQ + baseline questionnaire Validated a priori	Fertility diet	Rate of female factor infertility/ovulatory disorder infertility	-
Gaskins et al., 2014 [44]	USA	Cohort study	11,072 women 15,950 pregnancies Natural Conception	Self-reported FFQ + baseline questionnaire Validated a priori	Alternative Healthy Eating Index 2010 Alternative Mediterranean diet Fertility diet	Pregnancy loss (miscarriages and stillbirths)	-
Hartman et al., 2021 [45]	USA	Cohort study	131 couples Natural Conception	Three separate telephone 24-h dietary recalls a priori	Low energy density diet	Clinical pregnancy	-

ART: Assisted reproductive techniques, FFQ: Food frequency questionnaire, RBC: Red blood cell, tHcy: Total homocysteine, FSH: Follicle stimulating hormone, LH: Luteinizing hormone, AMH: Anti mullerian hormone, HCG: Human chorionic gonadotropin, AFC: Antral follicle count, ET: Embryo transfer.

**Table 3:** ROBINS–I tool, Risk Of Bias In Non-Randomised Studies of Interventions.

ROBINS-I Observational Studies									
Risk of Bias Domains									
		D1	D2	D3	D4	D5	D6	D7	Overrall
Study	Vujkovic (2010) [14]	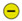	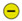	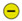	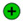	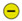	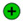	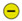	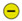
	Twigt (2012) [35]	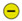	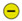	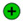	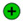	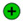	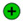	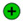	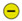
	Karayiannis (2018) [40]	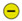	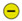	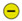	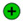	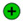	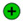	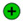	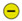
	Sugawa (2018) [41]	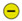	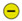	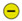	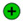	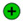	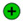	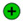	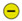
	Gaskins (2019) [21]	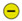	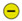	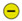	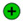	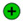	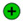	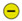	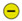
	Sun (2019) [42]	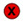	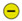	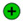	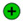	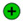	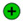	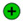	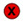
	Ricci (2019) [43]	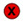	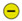	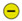	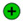	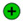	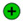	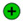	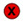
	Chavarro (2007) [15]	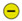	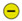	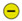	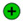	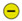	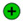	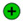	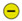
	Toledo (2011) [46]	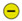	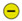	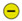	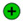	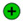	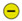	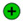	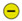
	Gaskins (2014) [44]	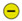	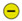	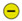	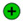	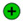	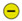	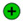	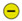
	Hartman (2021) [45]	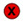	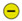	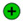	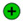	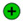	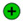	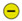	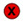

Domains: D1: Bias due to confounding. D2: Bias due to selection of participants. D3: Bias in classification of interventions. D4: Bias due to deviations from intended interventions. D5: Bias due to missing data. D6: Bias in measurement of outcomes. D7: Bias in selection of the reported result. Judgement: 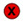 Serious. 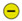 Moderate. 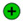 Low.

**Table 4:** Results of primary outcomes across included studies.

Author	Diet	Biochemical Pregnancy	Clinical Pregnancy	Live Births	Infertility Rates	Pregnancy Loss	Adjusted for Confounders	Confounders Controlled (Confounders Measured but Not Controlled)
Vujkovic et al., 2010 [14]	Mediterranean	OR 1.27 (95% CI 0.48–3.39) ^a^	-	-	-	-	OR 1.4 (95% CI 1.0–1.9) (Biochemical pregnancy) (Couple adherence) ^a^	Age, BMI, smoking, alcohol, IVF/ICSI treatment, stimulation scheme
	Health conscious–low processed	OR 0.68 (95% CI 0.27–1.76) ^a^	-	-	-	-	OR 0.8 (95% CI 0.6–1.0) (Biochemical pregnancy) (women adherence) ^a^	
Twigt et al., 2012 [35]	Dutch dietary guidelines	-	OR 0.66 (95% CI 0.29–1.48) ^b^	-	-	-	OR 2.94 (95% CI 0.88–9.76) (Clinical pregnancy) ^b^	Age, smoking, BMI, partner diet, treatment indication (Alcohol, exercise, stress, ethnicity, education)
Karayiannis et al., 2018 [40]	Mediterranean	OR 1.98 (95% CI 1.05–3.78) ^a^	OR 2.43 (95% CI 1.28–4.63) ^a^	OR 2.64 (95% CI 1.37–5.07) ^a^	-	-	RR 3.12 (1.40–7.10) (Live births) ^a^	Age, stimulation scheme, BMI, energy intake, treatment indication, anxiety, supplements
Sugawa et al., 2018 [41]	Vegetables and seafood	-	OR 0.88 (95%CI 0.32–2.38) ^a^	-	-	-	OR 0.90 (95% CI 0.3–2.69) (Clinical pregnancy) ^a^	Age, BMI, parity, education, smoking, alcohol, folate (Number of oocytes retrieved, Veeck’s criteria)
	Rice and Miso	-	OR 0.81 (95%CI 0.22–2.93) ^a^	-	-	-	OR 0.72 (95% CI0.18–2.93) (Clinical pregnancy) ^a^	
	Western	-	OR 0.70 (95% CI 0.21–2.28) ^a^	-	-	-	OR 0.84 (95% CI 0.23–3.11) (Clinical pregnancy) ^a^	
Gaskins et al., 2019 [21]	Mediterranean diet	RR 1.12 *p* = 0.17 ^a, c^ OR 1.26 ^d^	RR 1.12 *p* = 0.25 ^a, c^ OR 1.24 ^d^	RR 1.32 *p* = 0.06 ^a, c^ OR 1.55 (95% CI 0.99–2.42) ^b^	-	-	OR 1.54 (95% CI 0.85–2.80) (Live births) ^b^	Age, BMI, calorie intake, smoking, vigorous exercise
	Alterative Healthy Eating Index 2010	RR 0.87 *p* = 0.12 ^a, c^ OR 0.72 ^d^	RR 0.82 *p* = 0.08 ^a, c^ OR 0.67 ^d^	RR 0.84 *p* = 0.19 ^a, c^ OR 0.72 (95% CI 0.45–1.14) ^b^	-	-	OR 0.72 (95% CI 0.40–1.30) (Live births) ^b^	
	Fertility diet	RR 1.00 *p* = 0.83 ^a, c^ OR 1.00 ^d^	RR 0.98 *p* = 0.89 ^a, c^ OR 0.96 ^d^	RR 1.16 *p* = 0.37 ^a, c^ OR 1.27 (95% CI 0.79–2.05) ^b^	-	-	OR 1.36 (95% CI 0.75–2.50) (Live births) ^b^	
	ProFertility diet	RR 1.48 *p* =< 0.001 ^a, c^ OR 2.51 ^d^	RR 1.53 *p* =< 0.001 ^a, c^ OR 2.37 ^d^	RR 1.70 *p* =< 0.001 ^a, c^ OR 2.14 (95% CI 1.35–3.40) ^b^	-	-	OR 2.56 (95% CI 1.42–4.63) (Live births) ^b^	
Sun et al., 2019 [42]	Mediterranean	-	OR 0.72 (95% CI 0.38–1.35) ^a^	-	-	-	None provided	Age, BMI, duration of infertility (Infertility diagnosis, sperm concentration, total motile sperm basal FSH, Gn duration, dosage Gn)
Ricci et al., 2019 [43]	Mediterranean	-	-	OR 1.00 (95% CI 0.58–1.73) ^a^	-	-	OR 0.91 (95% CI 1.63–0.54) (Live births) ^d^	Age, BMI, previous ART, leisure physical activity, smoking, caloric intake, calorie restriction
Toledo et al., 2011 [46]	Mediterranean	-	-	-	OR 0.56 (95% CI 0.35–0.95) ^a^	-	OR 0.74 (95% CI 0.55–1.00) (Infertility) ^a^	Age, BMI, smoking, physical activity, alcohol, total energy intake, supplement use, intake of plant proteins, animal proteins, trans fat, fibre
	Western	-	-	-	OR 0.83 (95% CI 0.64–1.08) ^a^	-	OR 0.91 (95% CI 0.66–1.24) (Infertility) ^a^	
Chavarro et al., 2007 [15]	Fertility diet	-	-	-	-	-	RR 0.32 (95% CI 0.23–0.48) (Ovulatory infertility) RR 0.73 (95% CI 0.57–0.95) (All cause infertility)	Age, BMI, alcohol, coffee, smoking, physical activity, parity, COCP use
Gaskins et al., 2014 [44]	Alternative Mediterranean diet	-	-	-	-	RR 1.11 (95% CI 1.01–1.23) ^a^	RR 1.02 (95% CI 0.98, 1.05) (Pregnancy loss) ^a^	Age, BMI, total energy, smoking, physical activity, history of infertility, marital status, race, nulliparity
	Alternative Healthy Eating Index 2010	-	-	-	-	RR 1.23 (95% CI 1.13–1.36) ^a^	RR 1.01 (95% CI 0.98–1.05) (Pregnancy loss) ^a^	
	Fertility diet	-	-	-	-	RR 0.91 (95% CI 0.82–1.00) ^a^	RR 0.98 (95% CI 95–1.01) (Pregnancy loss) ^a^	
Hartman et al., 2021 [45]	Low energy density diet	-	OR 1.44 (95% CI 0.62–3.36) ^a^	OR 1.44 (95% CI 0.62–3.34) ^a^	-	-	OR 2.56 (live birth) ^a, e^	Race, partner diet, physical activity, BMI, total energy intake (Educational status, income, female smoking, total trans fats, total protein, alcohol)
	High energy density diet	-	OR 0.69 (95% CI 0.30–1.61) ^a^	OR 0.69 (95% CI 0.30–1.61) ^a^	-	-	OR 0.39 (Live birth) ^a, e^	

OR: odds ratio, RR: relative risk, CI: confidence interval, BMI: Body mass index, IVF: In Vitro Fertilization, ICSI: intracytoplasmic sperm injection, FSH: Follicle stimulating hormone, Gn: Gonadotropin, COCP, Combined Oral Contraception Pill. ^a^ = Original published results. ^b^ = Additional information provided by author. ^c^ = Adjusted RR, *p* value for trend across four quartiles. (Crude RR, CI, and crude OR not published). ^d^ = Converted from adjusted RR. ^e^ = CI not published.

**Table 5:** Subgroup analysis, Mediterranean diet.

Subgroup	Number of Studies	OR (95% CI)	Heterogeneity	
			*I*^2^ (%)	*p*
Primary Outcome				
Biochemical pregnancy	1	1.27 (0.48–3.39)	-	-
Clinical pregnancy	1	0.72 (0.38–1.35)	-	-
Live birth	3	1.51 (0.88–2.62)	67	0.05
Dietary measurement method				
a priori	4	1.27 (0.76–2.14)	70	0.02
posteriori	1	1.27 (0.48–3.39)	-	-

OR, Odds Ratio.

## Data Availability

All data are provided in the journal as Supplementary Material, but additional requirements can be made through contact with the authors.

## Supplementary Materials

The following supporting information can be downloaded at: https://www.mdpi.com/article/10.3390/nu15112589/s1, Supplementary file: Supplementary Figures.
