# Supplementary material for: Can Dietary Patterns Impact Fertility Outcomes? A Systematic Review and Meta-Analysis
Source: Nutrients. 2023 May 31;15(11):2589. doi: 10.3390/nu15112589 (PMC10255613; doi:10.3390/nu15112589)
Supplement: Supplementary file 1 [file nutrients-15-02589-s001.zip › Supplementary Figures.pdf]

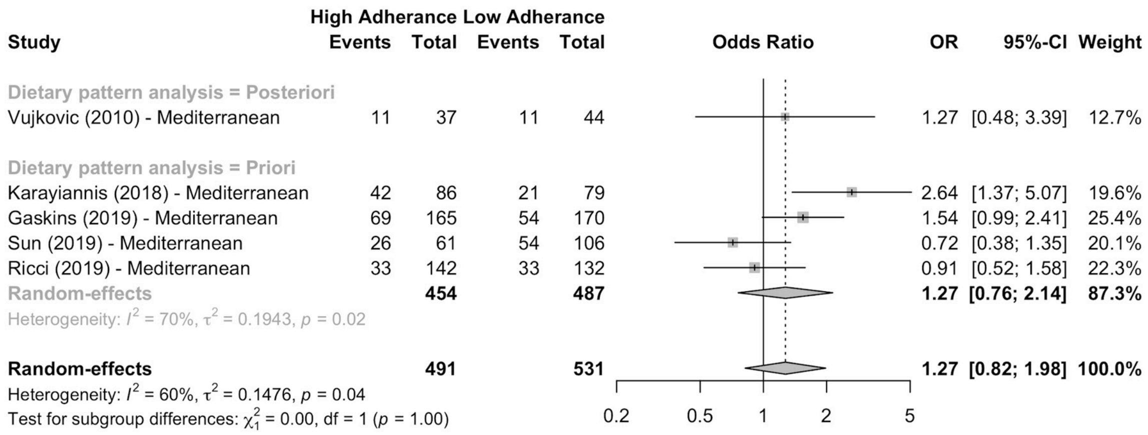

Figure S1: Crude OR of the association of Mediterranean diet to ART outcomes by subgroup: dietary pattern analysis (*a priori*, *posteriori*)

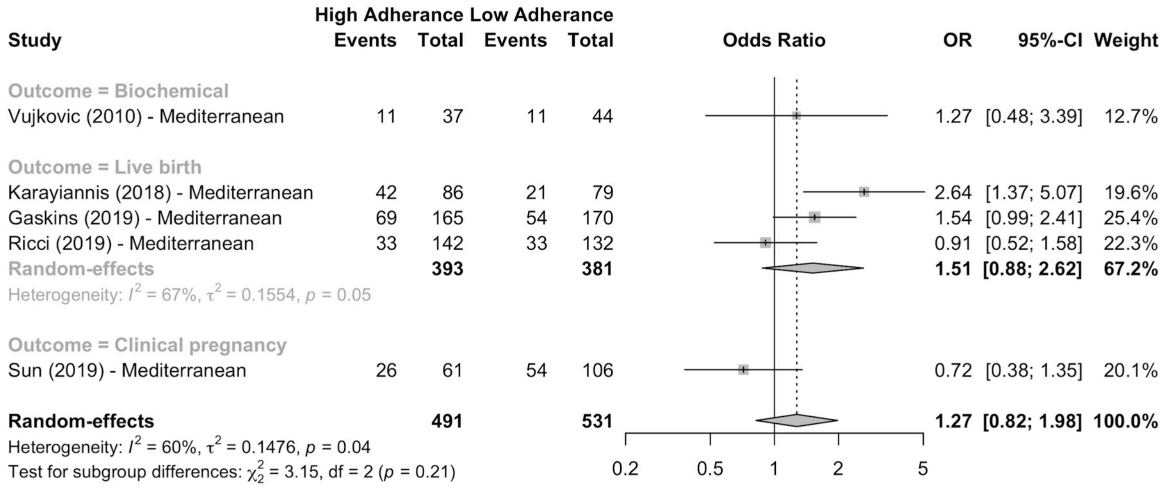

Figure S2: Crude OR of the association of Mediterranean diet to ART outcomes by subgroup: ART outcome (biochemical pregnancy, clinical pregnancy, live birth)

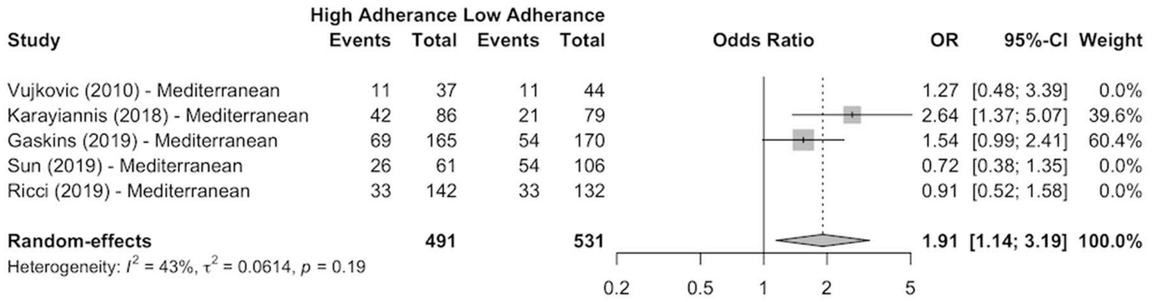

Figure S3: Crude OR of the association of Mediterranean diet to ART outcomes, sensitivity analysis: excluding high risk-of-bias studies

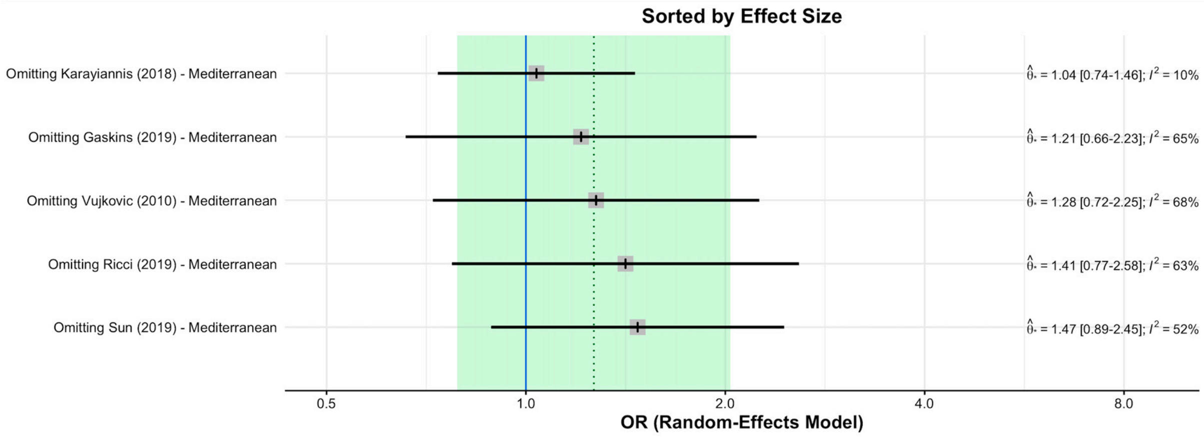

Figure S4: Crude OR of the association of Mediterranean diet to ART outcomes: Leave-one-out analysis

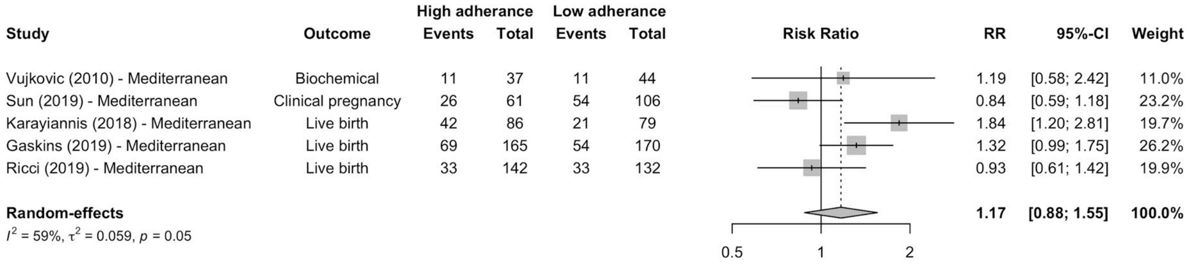

RR, relative risk. ART. CI, confidence interval. τ2, tau-squared

Figure S5: Crude RR of the association of Mediterranean diet to ART outcomes

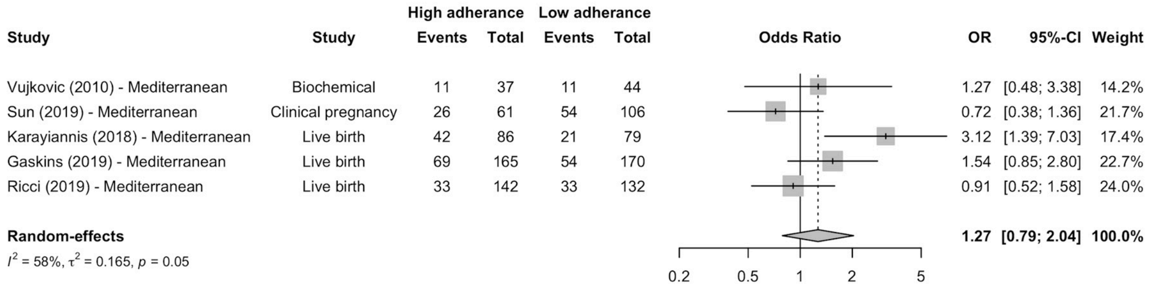

Figure S6: Adjusted OR of the association of Mediterranean diet to ART outcomes

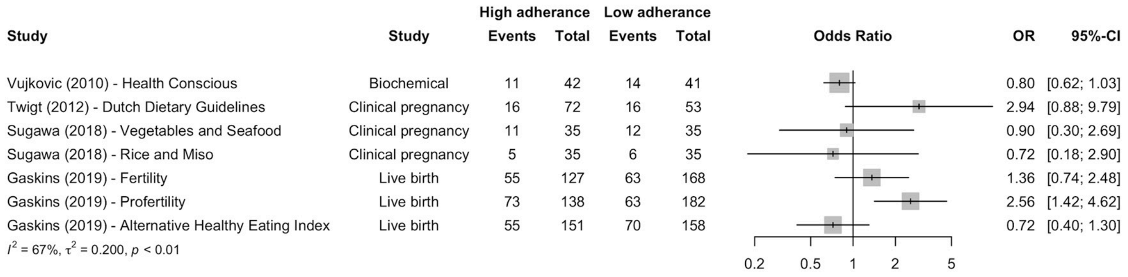

Figure S7: Adjusted OR of the association of Healthy diets and ART outcomes
