# Supplementary material for: Can Dietary Patterns Impact Fertility Outcomes? A Systematic Review and Meta-Analysis
Source: Nutrients. 2023 May 31;15(11):2589. doi: 10.3390/nu15112589 (PMC10255613; doi:10.3390/nu15112589)
Supplement: Supplementary file 1 [file nutrients-15-02589-s001.zip › Supplementary Material.pdf]

Registered in Open Sciences Framework (10.17605/OSF.IO/FBV6W)

Search strategy
Search date: 27 September 2021
Data bases:

- Ovid Medline
- EMBASE
- CAB Direct
- CINAHL Plus

Search strategy

1. preconcep$.tw.
2. pre-concep$.tw.
3. prepregnan$.tw.
4. pre-pregnan$.tw.
5. pregestation$.tw.
6. pre-gestation$.tw.
7. periconception$.tw.
8. peri-conception$.tw.
9. peri concpeiton$.tw.
10. interpregnan$.tw.
11. inter-pregnan$.tw.
12. intergestaion$.tw.
13. inter-gestation$.tw.
14. interconcp$.tw.
15. inter-concep$.tw.
16. internatal.tw.
17. inter-natal.tw.
18. before pregnan$.tw.
19. pregnancy intention.tw.
20. pregnancy plan$.tw.
21. reproductive age*.tw.
22. exp Preconcpeiton Care/
23. exp family planning service/
24. exp perinatal care/
25. exp Infertility, Female/
26. subfertil$.tw.
27. infertil$.tw.
28. fertility.tw
29. fecund$.tw.
30. in vitro fertili*ation.tw.
31. intrauterine insemination.tw.
32. artificial insemination.tw.
33. ivf.tw.
34. icsi.tw.
35. iui.tw.
36. ART.tw.
37. Embryo transfer.tw.
38. Assisted reproduc$.tw.

39. Assisted conce$.tw.
40. Artificial reproduc$.tw.
41. or/1-40$.tw.
42. diet$.tw
43. nutrition$.tw.
44. nutrient$.tw.
45. food$.tw.
46. eat$.tw.
47. lifestyle.tw.
48. life style.tw.
49. intake.tw.
50. consum$.tw.
51. meat.tw.
52. poultry.tw.
53. chicken.tw.
54. fish.tw.
55. seafood.tw.
56. soy.tw.
57. legume$.tw.
58. bean$.tw.
59. nut$.tw.
60. seed$.tw.
61. dairy.tw.
62. fruit.tw.
63. vegetable.tw.
64. cereal.tw.
65. grain$.tw.
66. core food.tw.
67. non-core food.tw.
68. discretionary food.tw.
69. non-discretionary food.tw.
70. sugar sweetened.tw.
71. soft drink$.tw.
72. juice.tw.
73. processed food$.tw.
74. feeding behavior/
75. exp food/
76. exp diet/
77. exp diet therapy/
78. exp Life style
79. or/42-78
80. 41 and 79
81. limit 79 to humans
